# Phylogenetic analysis of Shiga toxin 1 and Shiga toxin 2 genes associated with disease outbreaks

**DOI:** 10.1186/1471-2180-7-109

**Published:** 2007-12-04

**Authors:** James E Lee, Junelina Reed, Malcolm S Shields, Kathleen M Spiegel, Larry D Farrell, Peter P Sheridan

**Affiliations:** 1 Department of Biological Sciences, Idaho State University, 921 South 8^th ^Ave., Pocatello, ID 83209-8007, USA

## Abstract

**Background:**

Shiga toxins 1 and 2 (Stx1 and Stx2) are bacteriophage-encoded proteins that have been associated with hemorrhagic colitis, hemolytic uremic syndrome and other severe disease conditions. Stx1 and Stx2 are genetically and immunologically distinct but share the same compound toxin structure, method of entry and enzymatic function.

**Results:**

Phylogenetic analysis was performed using Stx1 and Stx2 amino acid and nucleotide sequences from 41 strains of *Escherichia coli*, along with known *stx *sequences available from GenBank. The analysis confirmed the Stx1 and Stx2 divergence, and showed that there is generally more sequence variation among *stx2 *genes than *stx1*. The phylograms showed generally flat topologies among our strains' *stx1 *and *stx2 *genes. In the *stx2 *gene, 39.5% of the amino acid sites display very low nonsynonymous to synonymous substitution ratios.

**Conclusion:**

The *stx1 *and *stx2 *genes used in this phylogenetic study show sequence conservation with no significant divergence with respect to place or time. These data could indicate that Shiga toxins are experiencing purifying selection.

## Background

Shiga toxin was discovered in *Shigella dysenteriae *serotype 1 by Kiyoshi Shiga in 1898 [1]. The cytotoxic effects of *Escherichia coli*-produced Shiga toxin on Vero cells were first described 29 years ago [2]. A few years later, the toxin was closely associated with hemorrhagic colitis, hemolytic uremic syndrome (HUS) and other severe disease conditions [3,4]. Shiga toxin producing *E. coli *have been implicated in food borne, waterborne and airborne outbreaks in studies all over the world [5-7]. Much of the focus of identification and characterization of Shiga toxin has been on *E. coli *O157:H7 strains, even though many cases of Shiga toxin associated disease were caused by other serotypes of *E. coli *[8]. The toxin has also been observed in other bacterial genera, including *Citrobacter*, *Enterobacter *and *Acinetobacter *[9-11].

Shiga toxin 1 (Stx1) and Shiga toxin 2 (Stx2) are encoded on a lambdoid bacteriophage. Stx1 is genetically and immunologically distinct from Stx2, showing 55–60% genetic and amino acid identity [12]. Stx1 is very similar to the Shiga toxin (Stx) found in *Shigella dysenteriae *type 1 [13]. Several variants of Stx1 (Stx1c and Stx1d) and Stx2 (Stx2c, Stx2d, Stx2e, Stx2f, and Sxt2g) have been described [14-17]. Both Stx1 and Stx2 are compound toxins made up of one 32 kDa A subunit and five identical 7.7 kDa B subunits[18,19]. The B subunits form a pentameric hollow ring that encircles the carboxyl end of the A amino acid chain. The B subunits of Stx1, and most Stx2 type toxin molecules bind to specific glycosphingolipid globotriaosylceramide (Gb3) receptors in eukaryotic cell membranes. Stx2e B subunits preferentially bind to globotetraosylceramide (Gb4) [20], which allows the toxin to target different cell types. Once bound, receptor mediated endocytosis produces toxin-containing vesicles that travel through the Golgi apparatus and endoplasmic reticulum. The A subunit is then proteolytically cleaved into A1 (27.5 kDa) and A2 (4.5 kDa), but A1 and A2 remain covalently connected through a disulfide bond between two cysteine residues. When the cysteines are reduced, the catalytically active A1 enzyme cleaves a specific adenine from the 28S rRNA of the 60S ribosomal subunit [21]. Without this adenine, the GTP/elongation factor Tu/amino acyl-tRNA complex is unable to associate properly with the ribosome. Amino acid chain elongation is stopped, usually resulting in cell death.

Shiga toxin was shown to have the same N-glycosidase depurinating enzymatic function and active site conformation found in another ribosomal inhibiting protein (RIP), ricin [22,23]. Comparison of the crystal structures of Stx from *Shigella dysenteriae *[19] and Stx2 from *E. coli *[24] showed that the active sites were similar to that of ricin. Both ricin and Shiga toxin are considered type II RIPs because of the lectin property of the B subunit that allows the enzymatic A subunit to be internalized for access to the ribosome. Abrin, modeccin, volkensin and viscumin are examples of other RIPs that use the same method of entry and mechanism of action [25-28].

Despite their similarities, Stx1 and Stx2 produce different degrees and types of tissue damage. Enterohemorrhagic *E. coli *that produce Stx2 are more likely to cause hemolytic uremic syndrome than are Stx1 producers [29]. This could be due to accessibility of the active site, differences in the carboxyl end of the A subunit, or differences in binding affinities of the B subunit pentamer to Gb3 [24].

Other studies have shown how different types of pathogenic *E. coli *and *Shigella *have developed using multilocus sequence typing (MLST) of several housekeeping genes [30-32], genomic hybridization by microarray [33] and comparative genomic sequencing by microarray [34]. Since Shiga toxin is encoded on a mobilizable bacteriophage, we focused on the phylogenetic diversity of the gene rather that the bacterium that happens to carry it. In this study we compared Shiga toxin gene nucleotide and amino acid sequences from twenty nine reference strains from the National Food Safety & Toxicology Center at Michigan State University and twelve clinical isolates from the Idaho Department of Health that contained one or both Stx genes. The *E. coli *strains used are both temporally and spatially distinct (Table 1). Included in the analysis were unique Shiga gene sequences that were available from Genbank. We confirmed that Stx2 shows more sequence variation than Stx1 among our reference and clinical isolates. We also showed that our isolates' *stx1 *and *stx2 *genes each respectively showed little divergence and may display purifying selection.

**Table 1:** *E. coli *reference strains used in this study. Information for the strains obtained from the National Food Safety & Toxicology Center at Michigan State University was taken directly from their web site.

**Michigan State Name**	**Name Used in This Study**^1^	**Shiga Toxin Gene**	**GenBank Accession Number**	**O antigen**	**H antigen**	**Host**	**Location**	**Date**
EDL933		*stx1*		157	7	Food	Michigan	1982
		*stx2*						
93–111	E12	*stx1*	EF441572	157	7	Human	Washington	1993
		*stx2*	EF441599					
OK-1	E13	*stx1*	EF441573	157	7	Human	Japan	1996
		*stx2*	EF441600					
86-24	E14	*stx2*	EF441601	157	7	Human	Washington	1986
2886-75	E15	*stx1*	EF441574	157	7	Human	California	1975
493/89	E16	*stx2*	EF441602	157	-	Human	Germany	1989
E32511	E17	*stx2*	EF441603	157	-	Human	No Data	1985
G5101	E18	*stx1*	EF441575	157	7	Human	Washington	1995
		*stx2*	EF441604					
5905	E19	*stx2*	EF441605	55	7	Food	No data	1994
DEC8B	E22	*stx1*	EF441576	111	8	Human	Idaho	1986
		*stx2*	EF441606					
3007-85	E23	*stx1*	EF441577	111	-	Human	Nebraska	1985
		*stx2*	EF441607					
TB226A	E24	*stx2*	EF441608	111	-	Human	Washington	1991
928/91	E25	*stx1*	EF441578	111	-	Human	Germany	1991
		*stx2*	EF441609					
412/55	E26	*stx1*	EF441579	111	No data	Human	Germany	1955
DEC8C	E27	*stx1*	EF441580	111	-	Calf	Scotland	No data
C412	E28	*stx1*	EF441581	111	No data	Calf	S. Dakota	1986
H19	E29	*stx1*	EF441582	26	11	Human	Canada	1977
DEC10B	E210	*stx1*	EF441583	26	11	Human	Australia	1986
TB285C	E213	*stx1*	EF441584	26	-	Human	Washington	1991
BCL19	E216	*stx1*	EF441585	No data	-	Cow	California	No data
DEC10J	E217	*stx1*	EF441586	70	11	Human	Canada	1988
ED-31	E218	*stx1*	EF441587	111	-	Human	Italy	No data
		*stx2*	EF441610					
CL-3	S1	*stx2*	EF441618	113	21	Human	Canada	1980
G5506	S3	*stx2*	EF441619	104	21	Human	Montana	1994
B2F1	S4	*stx2*	EF441620	91	21	Human	Canada	1985
TB154A	S7	*stx1*	EF441595	103	6	Human	Washington	1991
88–1509	S8	*stx1*	EF441596	15	27	Human	Canada	1988
		*stx2*	EF441621					
M2113	S10	*stx1*	EF441597	156	21	Cow	Germany	No data
BCL17	S11	*stx1*	EF441598	5	No data	Cow	California	No data
90–1787	S12	*stx2*	EF441622	X03	-	Cow	Canada	No data

^1^These names used only in the alignments.

## Results and Discussion

### Similarity of Shiga Toxin 1 A and B subunit genes

**Figure 1:**
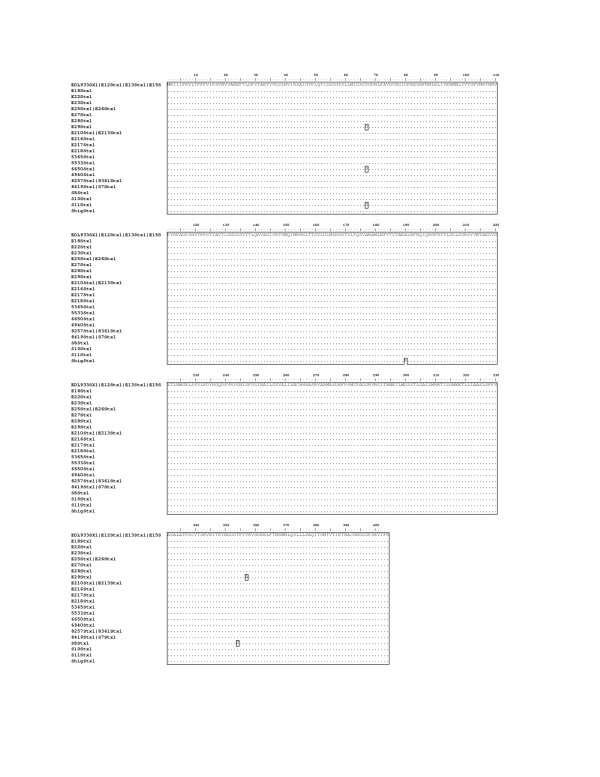
**Stx1 A and B Subunit Amino Acid Alignment**. Sequences that are identical are on the same line and are pipe-delimited.

The Shiga Toxin 1 A and B subunit genes showed very little difference in amino acid sequence (Figure 1). There were only four amino acid differences among the *stx1 *genes in the entire alignment. The change from threonine to serine at A67 in strains H19, BCL17 and I6650 would predictably have little effect on the enzymatic activity of the A subunit. The B354 change from threonine to serine in 88–1509 and the B357 change from valine to alanine in H19 are also unlikely to change the binding function of the B subunit.

**Figure 2:**
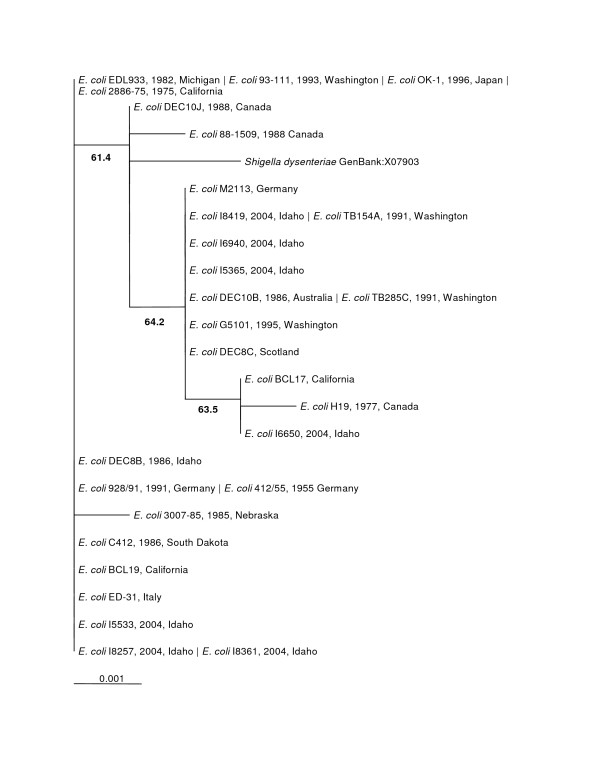
***stx1 *Maximum Likelihood Nucleotide Phylogenetic Tree (unrooted)**. Bootstrap values over 50% are displayed. Pipe-delimited strain names indicate identical sequences. Dates and locations of the isolates are shown if available. The horizontal bar shows 0.001 nucleotide substitutions per site.

**Figure 3:**
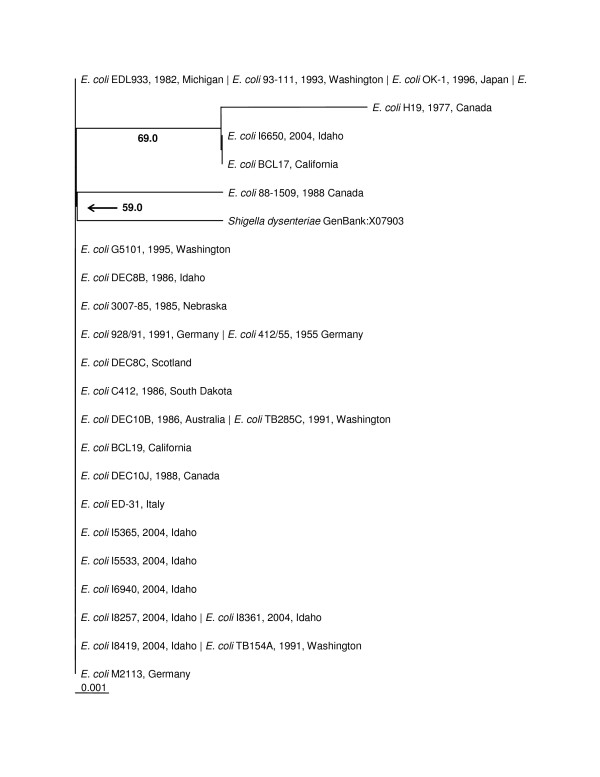
**Stx1 Distance Amino Acid Phylogenetic Tree (unrooted)**. Bootstrap values over 50% are displayed. Pipe-delimited strain names indicate identical sequences. Dates and locations of the isolates are shown if available. The horizontal bar shows 0.001 amino acid substitutions per site.

The phylogenetic trees made from the nucleotide sequences (Figure 2) and the amino acid sequences (Figure 3) were topologically the same with very short branch lengths. Bootstrap replicates in both trees supported the H19/BCL17/I6650 clade and the *Shigella dysenteriae*/88–1509 clade. The short branch lengths and relatively low bootstrap values can be directly attributed to the low number of informative characters in the analysis.

### Similarity of Shiga Toxin 2 A and B subunit genes

**Figure 4:**
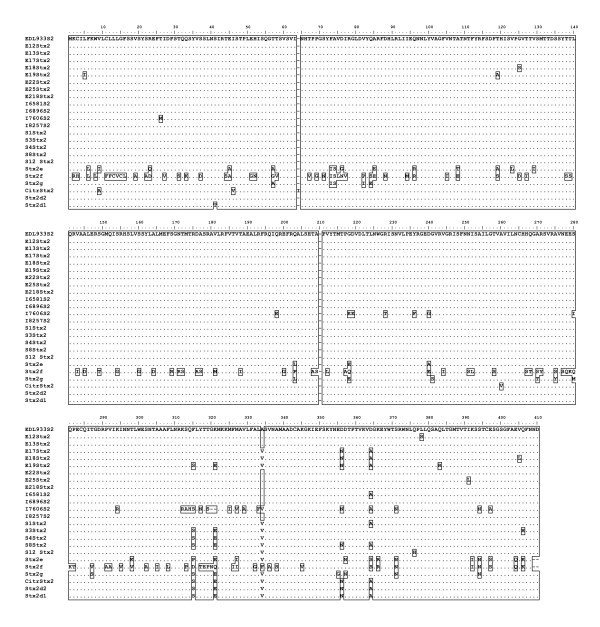
**Stx2 A and B Subunit Amino Acid Alignment**. Sequences that are identical are on the same line and are pipe-delimited.

The Shiga toxin 2 A and B subunit sequences showed much more sequence diversity than did the Stx1 group. None of the putative substitutions replaced key residues used in the active site [24] or either of the cysteines that form the disulfide bridge between A1 and A2. There were 56 total amino acid differences from EDL933 Stx2 in this study's sequences, 28 in the A subunit and 28 in the B subunit. Twenty-five of these changes occurred in I7606 Stx2. The number of positions in each subunit that were affected was 17 for the A subunit and 15 for the B subunit. The Stx2 alignment (Figure 4) shows all of the amino acid differences among this study's sequences, along with Stx2d1, Stx2d2, Stx2e, Stx2f, Stx2g and *Citrobacter freundii *Stx2 sequences from GenBank.

Strains E32511 and B2F1 have each been shown to contain two copies of the *stx2 *gene [35,36]. E32511 carries one *stx2 *gene that is almost identical to the EDL933 sequence and a second that differs by 15 nucleotides. Three of these nucleotide differences result in three amino acid changes in the B subunit only. The *stx2 *amplified from E32511 matches the Genbank accession number M59432 with no equivocal peaks in the chromatogram. B2F1 carries the *stx2d1 *and *stx2d2 *genes represented by the accessions AF479828 and AF479829, respectively. These sequences differ from EDL933 *stx2 *by 10 nucleotides with one amino acid change. The sequencing reaction chromatogram from our *stx2 *amplicon of the B2F1 strain showed nucleotide differences between both *stx2d1 *and *stx2d2 *with no ambiguities. These differences resulted in three amino acid changes along the sequence. In both E32511 and B2F1, the few sequence differences did not affect our analysis. However, until further analysis of our E32511 and B2F1 strains is complete to determine whether they contain one or two *stx2 *genes, our sequences EF441603 and EF441620 should be held in question.

**Figure 5:**
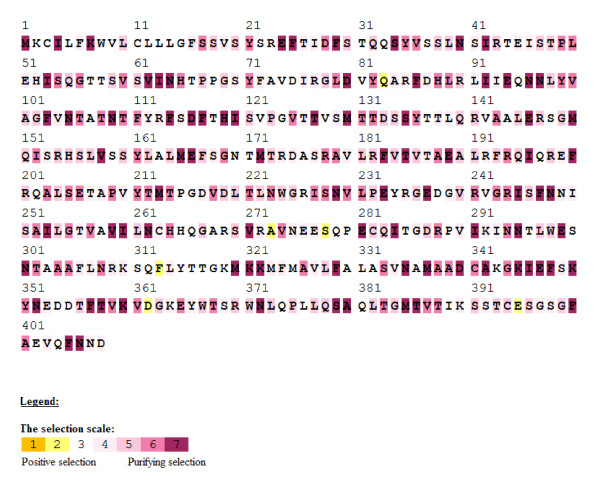
**Color Coded Selecton Results for *stx2 *Sequences**. Nonsynonymous:synonymous (d_N_:d_S_) ratios using the output from the Selecton program. Shades of yellow (1 and 2) indicate a d_N_:d_S _ratio of > 1 or positive selection. Any shade of bordeaux (4–7) indicates a d_N_:d_S _ratio of < 1 or purifying selection.

The ratio non-synonymous (d_N_) to synonymous (d_S_) substitutions was used to estimate whether positive or purifying selection had occurred at each amino acid site. A nonsynonymous:synonymous (d_N_:d_S_) ratio of greater than one indicates positive selection at that amino acid site whereas a d_N_:d_S _ratio of less than one shows purifying selection. Forty percent of the amino acid sites in the full alignment showed purifying selection using the Selecton program [37]. The Stx2 A and B subunits showed 37.3% and 44.9%, respectively, of their amino acid sites were well conserved. (Figure 5)

**Figure 6:**
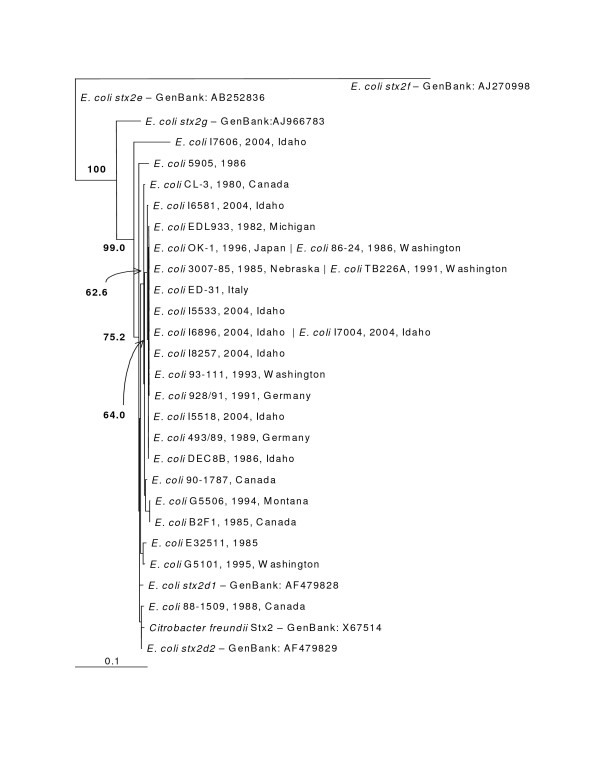
***stx2 *Maximum Likelihood Nucleotide Phylogenetic Tree (unrooted)**. Bootstrap values over 50% displayed. Pipe-delimited strain names indicate identical sequences. Dates and locations of the isolates are shown if available. The horizontal bar shows 0.1 nucleotide substitutions per site.

**Figure 7:**
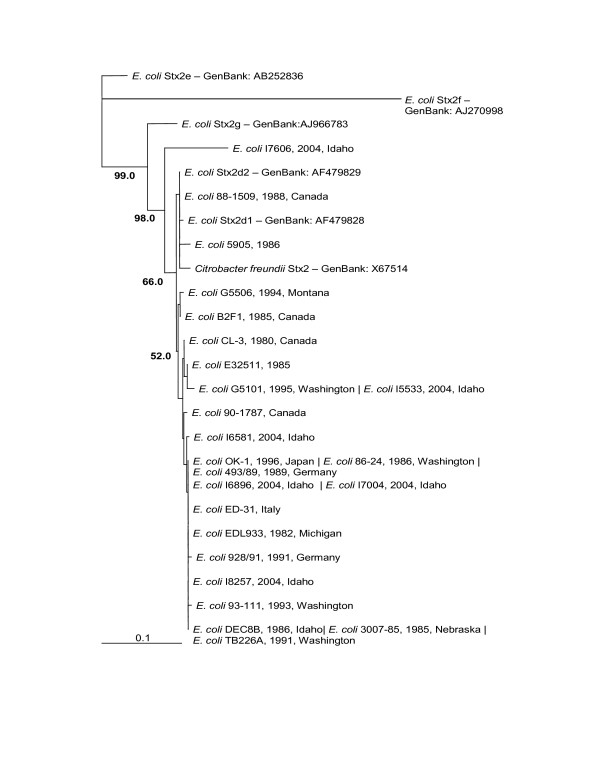
**Stx2 Distance Amino Acid Phylogenetic Tree (unrooted)**. Bootstrap values over 50% are displayed. Pipe-delimited strain names indicate identical sequences. Dates and locations of the isolates are shown if available. The horizontal bar shows 0.1 amino acid substitutions per site.

The nucleotide phylogenetic tree (Figure 6) and the amino acid phylogenetic tree (Figure 7) showed almost the same topology with short branch lengths for our strains. The obvious dissimilarity of Stx2e, Stx2f and Stx2g is striking in both the alignment and phylogenetic trees and is well supported by the high bootstrap values. Idaho strain I7606 had the most divergent sequence among the strains obtained from the Idaho State Health Department and is most closely related to Stx2d A subunit [38]. The lower bootstrap values in the figures are characteristic of highly similar sequences in the branches. Interestingly, the *C. freundii *Stx2 grouped with the Stx2d group of elastase activated Shiga toxins [39].

## Conclusion

**Figure 8:**
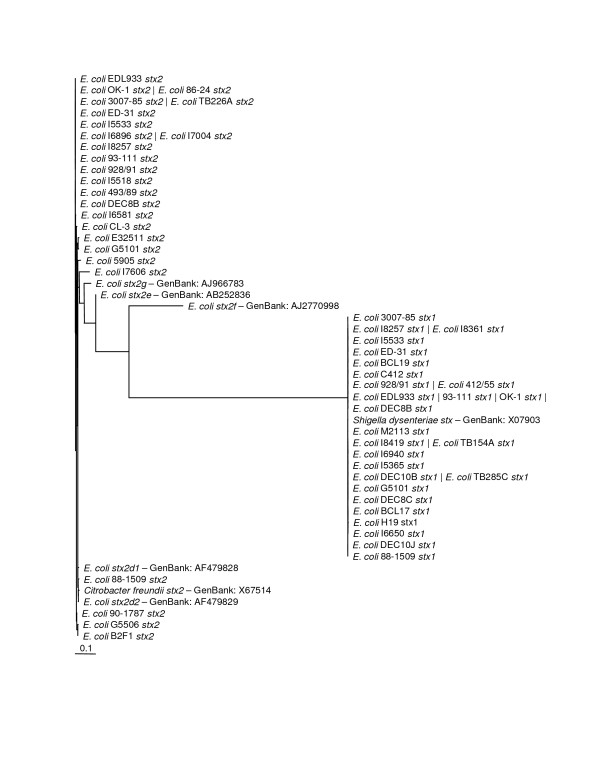
**Unrooted Maximum Likelihood Nucleotide Phylogenetic Tree of all Shiga Toxin Sequences**. The horizontal bar shows 0.1 nucleotide substitutions per site.

This investigation into the phylogenetic relationships among Shiga toxins confirmed that the two major groups, Stx1 and Stx2, are easily differentiated by both their amino acid and nucleotide sequences. The phylogram in Figure 8 shows *stx1 *and *stx2 *forming two distinct clades with *stx2f *branching off significantly from both. The *stx1 *group displayed an almost flat phylogeny whereas the *stx2 *group has much more sequence diversity. Stx2d, Stx2e and Stx2g all show at least 91% similarity to the EDL933 *stx2 *gene. The Stx2f amino acid and nucleotide sequences are only 72.5 and 70.4 percent similar to EDL933 Stx2 sequences, respectively, making it the most divergent Stx2 found to date. The greater diversity of Stx2 sequences could be the result of sampling bias. Since the majority of the gene sequences came from human outbreak pathogens and because Stx2 is associated with more severe disease, this could have an effect on what strains are recovered. Despite the fact that diarrheal illness is a leading causes of death worldwide [40], most sources providing isolates used in this study are located in areas with well established public health systems. This source location could also introduce bias into the isolates investigated.

The significance of compiling these sequences became clear when both the Stx1 tree and Stx2 tree showed generally flat topologies. Our further analysis of the nucleotide sequences for Stx2 A and B subunits showed that a large proportion of the amino acid sites had undergone purifying selection. Our hypothesis when undertaking this project was that Shiga toxin sequences from different places and collected at different times would show divergence. Our data analysis indicates that there is no significant divergence among either the Stx1 or Stx2 gene sequences based on collection time or location. The Stx1 phylogeny (Figure 2 and 3) shows that these genes do not vary greatly and are perhaps undergoing purifying selective pressure. The Stx2 phylogeny (Figure 4 and 5) is also generally flat among the strains we tested. This would indicate that among our strains, both the Stx1 and Stx2 gene sequences are closely conserved. One reason for the conservation of this sequence could be that intestinal Shiga toxin producing *E. coli *in the bovine host are mitigating Bovine Leukemia Virus induced disease [41]. Another recent finding suggests that the Shiga toxin might play a role in helping *E. coli *survive predation from bacteriovores outside of the cow [42]. Since many of our strains can be related to illness produced from bovine feces, it is possible that a Shiga toxin gene that is beneficial to the bacterium that carries it has undergone purifying selection to the benefit of both their bacterial and mammalian host.

## Methods

### Culture and Colony Isolation

Forty-one strains of *Escherichia coli *were used, including 29 outbreak reference strains provided by the National Food Safety & Toxicology Center at Michigan State University, and twelve clinical isolates from patients reported to the Idaho Department of Health. The characteristics and sources of *E. coli *strains used are described in Table 1. The specimens were received as 25% glycerol stocks on wet ice. *E. coli *strains were grown at 37°C in Luria-Bertani (LB) broth and isolated on LB agar.

### DNA Extraction

After isolation on LB agar, each strain was grown in 11 ml of LB broth for 18–24 hours at 37°C with shaking. All DNA extractions were performed using the PureGene DNA Isolation Kit (Gentra Systems, Minneapolis, MN). To maximize the yield of DNA, the following modifications to the PureGene protocol were made: 1) The time for cell lysis was increased to 10 minutes; 2) The DNA was precipitated using 300 μl 100% Isopropanol (tubes were inverted 50 times, incubated at -20°C for 1 hour and then centrifuged for 5 minutes at 13–16,000 × g); 3) DNA was dissolved in 100 μl sterile filtered 10 mM Tris, pH 8.3.

### Amplification of *Stx *genes

Primer sequences targeting specific *stx1 *and *stx2 *genes are listed in Table 2, along with PCR conditions used. The *stx1 *primers amplified 1178 bp of the 1227 bp sequence. The *stx2 *primers amplified the entire 1241 bp sequence. PCR master mix was made to final concentration of 1 × ThermoPol Reaction Buffer (New England BioLabs, Beverly, MA), 500 μM dNTP's (New England BioLabs), 0.02 units/μl Vent exo(-) polymerase (New England BioLabs), and 1 μM of each of the *stx1 *primers or *stx2 *primers. Five microliters of each amplification reaction was analyzed by 1% agarose gel electrophoresis. All gels were run at 80–90 V for 90 minutes in 1 × TAE buffer. Both gel and buffer contained 0.5 mg/ml ethidium bromide.

**Table 2:** Idaho *E. coli *strains used in this study. Information on the Idaho strains was obtained from the Idaho Department of Health.

**Strain Name**	**Shiga Toxin Gene**	**GenBank Accession Number**	**O antigen**	**H antigen**	**Host**	**Location**	**Date**
I5365	*stx1*	EF441588	26	11	Human	Idaho	2004
I5518	*stx2*	EF441611	121	19	Human	Idaho	2004
I5533	*stx1*	EF441589	157	-	Human	Idaho	2004
	*stx2*	EF441612					
I6581	*stx2*	EF441613	157	7	Human	Idaho	2004
I6650	*stx1*	EF441590	No data	No data	Human	Idaho	2004
I6896	*stx2*	EF441614	145	-	Human	Idaho	2004
I6940	*stx1*	EF441591	145	-	Human	Idaho	2004
I7004	*stx2*	EF441615	157	7	Human	Idaho	2004
I7606	*stx2*	EF441616	146	21	Human	Idaho	2004
I8257	*stx1*	EF441592	157	7	Human	Idaho	2004
	*stx2*	EF441617					
I8361	*stx1*	EF441593	111	-	Human	Idaho	2004
I8419	*stx1*	EF441594	103	25	Human	Idaho	2004

### Purification and Sequencing of Amplification Products

Amplicons were extracted from agarose gels using the QIAquick Gel Extraction Kit (Qiagen, Valencia, CA) according to the manufacturer's specifications. The amplicons were further purified for sequencing using Millipore Montage PCR filters (Billerica, MA). All sequencing was performed by the Idaho State University Molecular Research Core Facility using primers listed in Table 2. Accession numbers for nucleotide sequences submitted to GenBank are shown in Table 3.

**Table 3:** Primer sequences

Primer Name	Nucleotide Sequence 5'-3'	Target	PCR Conditions^a^		
	PCR Primers		Denature	Anneal	Extension
			
BGR1U	TCAACGAAAAATAACTTCGCTGAATCCC	Stx1 Base 1–28	95°C,	61°C,	72°C,
BGR1D	CAGTTAATGTGGTTGCGAAGGAATTTACC	Stx1 Base (1150–1178)	60 sec	60 sec	240 sec
					
BGR2U	ATGAAGTGTATATTATTTAAATGGGTACTGTG	Stx2 Base 1–32	95°C,	61°C,	72°C,
BGR2D	TCAGTCATTATTAAACTGCACTTCAG	Stx2 Base (1201–1226)	60 sec	60 sec	240 sec
					
	Sequencing Primers	Target			

Stx1SeqF	TGTAACCGCTGTTGTACCTGG	Stx1 Base (367–387)			
Stx1SeqR	TTAATACTGAATTGTCATCATCATGC	Stx1 Base 778–803			
Stx2SeqF	TTGCATTAGCTTCTGTTAATGCA	Stx2 Base 1000–1022			
Stx2SeqR	CATTCCGGAACGTTCCAG	Stx2 Base (433–450)			

^a ^PCR was performed for 30 cycles followed by a final extension step of 10 min at 72°C.

### Sequence Alignment and Phylogenetic Analysis

All amplicons were sequenced on both strands except the first and last 30 bases that were sequenced in only one direction in duplicate from internal primers. Raw sequence alignment was performed using Vector NTI 9 (InforMax, Invitrogen, Carlsbad, CA). Reference sequences for Shiga toxins used in the analysis were obtained from GenBank and are indicated in the phylogenetic tree by their accession numbers. Consensus sequences were entered into BioEdit 7.0.5.2 [43], aligned using ClustalW and then manually trimmed so that the nucleotide sequence accurately reflected the amino acid sequence. Amino acid sequence phylogenetic trees were created by distance analysis of the alignment using PAUP 4.0 Beta 10 [44]. Nucleotide phylogenetic trees were generated by maximum likelihood analysis of the alignment using PAUP 4.0 Beta 10 [44]. The Hasegawa-Kishino-Yano or HKY (*stx1*) and HKY with gamma likelihood models (*stx2*) were chosen after the data were analyzed using ModelTest [45] in PAUP. Construction of the nucleotide trees was done using the neighbor joining method with 1000 bootstrap replicates. The Selecton program [37] was used to determine the ratio of non-synonymous to synonymous substitutions. The top two purifying selection categories of 6 and 7 on the selection scale were used to calculate percentage of amino acids that were undergoing purifying selection. The maximum likelihood nucleotide phylogenetic tree produced above was inputted and the M8 evolutionary model [46] was used with EDL933 *stx2 *as the query sequence. Other evolutionary models available (M8a, M7, M5, MEC) produced results that were not significantly different. (data not shown) The full table of non-synonymous to synonymous ratios for *stx2 *sequences is available in additional file 1.

## Authors' contributions

JEL completed many of the amplifications, performed the alignments and phylogenetic analyses and drafted the manuscript. JR performed the majority of the amplifications, prepared the amplicons for sequencing, edited sequences and wrote portions of the methods. KMS and LDF participated in conceptual design of the study and helped to draft portions of the manuscript. MSS participated in design and coordination of the project and helped to revise portions of the manuscript. PPS participated in the study design, provided critical assistance with alignments and phylogenetic analyses, and helped draft portions of the manuscript. All authors read and approved the final manuscript.

## Supplementary Material

Additional file 1 Selecton Output. Selecton output file showing non-synonymous to synonymous ratio and confidence intervals of the *stx2 *sequences. Click here for file
